# Enhancing Health Policy Administration in LMICs: Dr. LJW Fellowship Program Insights (2021–2023)

**DOI:** 10.5334/aogh.4648

**Published:** 2025-06-18

**Authors:** Bomgyeol Kim, Yejin Kim, Jun Su Park, Soo Hyeok Choi, Su Hyun Kim, Vasuki Rajaguru, Hyejin Jung, Tae Hyun Kim

**Affiliations:** 1 Mo-Im Kim Nursing Research Institute, College of Nursing, Seoul, Republic of Korea; 2 Department of Public Health, Graduate School, Yonsei University, Seoul, Republic of Korea; 3 Department of Healthcare Management, Graduate School of Public Health, Yonsei University, Seoul, Republic of Korea; 4 Dr. LEE Jong-Wook Academy, Korea Foundation for International Healthcare, Seoul, Republic of Korea; 5 Department of Biohealth Industry Management, Graduate School of Transdisciplinary Health Sciences, Yonsei University, Seoul, Republic of Korea

**Keywords:** capacity building, training evaluation, Kirkpatrick’s model, health policy, KOFIH

## Abstract

*Background:* The Dr. LEE Jong-wook (LJW) Fellowship Program aims to enhance the capabilities of healthcare personnel in low- and middle-income countries (LMICs) through comprehensive training and education. This study evaluates the satisfaction and effectiveness of the Health Policy Administrator course within the program, focusing on participants from 2021 to 2023.

*Objective:* This study aims to assess the impact of the Dr. LJW Fellowship Program, specifically evaluating participants’ satisfaction, knowledge and competency improvement, and the adoption of learned knowledge in the workplace.

*Methods:* A mixed- methods study design was adopted, utilizing Kirkpatrick’s four-level evaluation framework to assess the program’s impact. A total of 39 public health policymakers from 19 LMICs participated in the training course at an affiliated university. The evaluation focused on training satisfaction, knowledge and competency improvement, competence achievement, and the practical adoption of learned knowledge. Descriptive statistics were used to analyze participant characteristics, while paired t-tests were employed to assess knowledge and competency improvement before and after the program.

*Results:* The program demonstrated high levels of participant satisfaction, with an overall satisfaction score of 92.9. Knowledge scores improved significantly, with an average increase of 61%, particularly in health statistics (77% improvement) and healthcare systems (56.3% improvement). Competency achievement was also high, with an average score of 92.5. However, the job adoption of learned knowledge scored lower, with supervisors and coworkers rating it at 70.9 and 72.1, respectively, indicating challenges in translating training into practical workplace applications.

*Conclusions:* The Dr. LJW Fellowship Program effectively enhanced participants’ knowledge and competencies in health policy administration. However, the lower scores in job adoption suggest a need for improved follow-up support and practical application strategies to ensure that the training’s benefits are fully realized in participants’ work environments.

## Background

*Official Development Assistance* (ODA) refers to the aid provided by governments and public institutions to promote economic development and social welfare in developing countries [1]. This assistance includes funding and technical cooperation extended to the governments, regions, and international organizations of these nations [1]. South Korea, one of the poorest nations at the time it joined the World Bank and International Monetary Fund in 1955, became a member of the Organization for Economic Cooperation and Development (OECD) in 1996 [2]. Furthermore, in 2000, South Korea was removed from the list of aid recipients by the OECD Development Assistance Committee (DAC), and in 2010, it transitioned to the 24th donor country [3]. This transition from an aid recipient to a donor nation is particularly noteworthy, representing the first such occurrence since the establishment of the OECD DAC [4]. As of 2022, South Korea’s ODA contributions totaled US$2.79 billion, ranking 16th among 31 DAC member countries [5].

In the realm of international development cooperation, there is growing emphasis on the training and education of personnel from recipient countries, underscoring the importance of invitational training in the ODA sector [6]. To address healthcare workforce education through sustained cooperation between developing and developed countries, continuous efforts are being made [7, 8]. Since the 2000s, the World Health Organization has formulated strategies for training and strengthening healthcare personnel [9].

The Korea Foundation for International Healthcare (KOFIH), a state agency administered by the Ministry of Health and Welfare, has been operating the Dr. LEE Jong-wook (LJW) School and coordinating the fellowship program since 2007 [10]. This program offers medium- to long-term invitational training for healthcare professionals, with the goal of enhancing the medical capabilities of healthcare personnel in partner countries, supporting the implementation of healthcare strategies in low- and middle-income countries (LMICs), and elevating the international recognition of South Korea’s advanced healthcare standards [10]. KOFIH will provide training to 1,500 healthcare professionals from 30 countries through this fellowship program from 2007 to 2023. By 2024, it plans to offer training to 215 healthcare professionals across 14 countries.

The Health Policy Administrator course within the Dr. LJW Fellowship Program targets key health policymakers and administrators working in central and regional health organizations in LMICs. While the short-term goal of the health policy course is to develop expertise in health policy and administration, its long-term objective is the transfer of knowledge, whereby participants apply what they have learned and disseminate this knowledge to their colleagues upon returning to their home countries and institutions [11]. To realize these objectives, the course managed participants’ learning progress and achievements, conducted numerous surveys, and held regular and ad hoc interviews to predict and evaluate the applicability of training in their home countries. The project team prepared and collected data using a survey to evaluate the efficacy of fellowship programs in fostering satisfaction, developing competencies, and enhancing practices. The importance of invitational training for personnel from developing countries is increasing, thus necessitating urgent research on this topic [12]. However, empirical studies on satisfaction with such training programs remain limited. Therefore, this study aimed to evaluate the satisfaction of participants in KOFIH’s Dr. LJW Fellowship Program training course for health policy administrators, providing foundational data to enhance the efficacy of invitational training and inform future programs.

## Methods

### Program outline

The Health Policy Administrator course within the Dr. LJW Fellowship Program targets key health policymakers and administrators working in central and regional health organizations in LMICs. This course aims to enhance the effectiveness of fellowship programs and expand future collaboration opportunities, ultimately improving residents’ health in partner countries.

**Figure 1:**
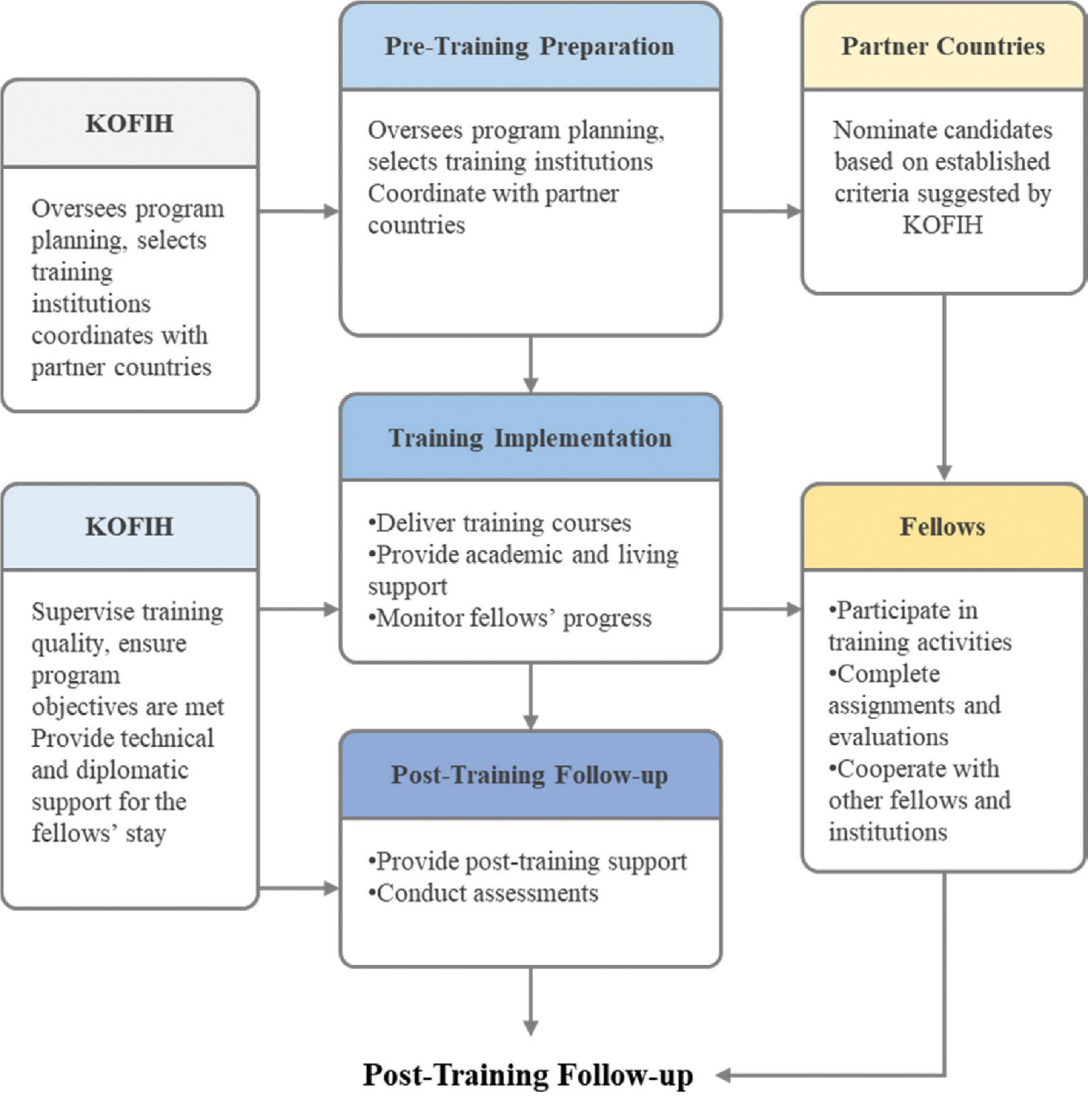
Dr. LEE Jong-wook Fellowship training program modules.

The selection of training institutions for the Dr. LJW Fellowship Program is conducted through a structured, merit-based, and competitive process designed to ensure both educational excellence and long-term programmatic sustainability. Institutions are assessed and selected based on their demonstrated expertise and operational capacity within their respective fields of specialization, utilizing Korea’s national competitive procurement platform. Notably, KOFIH implements this process through the Korean government’s procurement system, adhering to principles of transparency, fairness, and efficiency. Contracts are awarded for a period of three to four years, providing institutional continuity while upholding high standards of accountability and performance. The detailed process for selecting host institutions and fellows is presented in Supplementary File 1 and Figure 1.

The course commenced in 2007, when two Uzbekistanis received initial training, and since then, 169 fellows have participated in this training program from various institutions in South Korea. In terms of follow-up, four consecutive years (2021–2024) have been conducted at our affiliated university. The training program consisted of three modules: pre-training, training implementation (coursework, individual training, and field visits), and post-training management (Figure 1). The detailed instructions for the training program are presented in Supplementary Table 1.

Since 2021, the Dr. LJW Fellowship Program has trained public health professionals from LMICs through a structured and collaborative international program. Each year, 12 to 14 participants, selected from the Ministry of Health, regional or district-level health sectors, national research institutions, and primary healthcare services, engage in a 3-month training program in South Korea, followed by a 1-year post-training follow-up. Fellows from 19 countries participated in this study. Laos had the largest number of participants (20), followed by Ghana and Tanzania (18 each).

### Study design and participants

A mixed- methods design is adopted, focusing on quantitative and qualitative approaches. The development and assessment of this training course necessitate a modern, comprehensive evaluation approach. To enhance the reliability of training courses, it is crucial to implement a program and utilize an authentic performance evaluation model that employs an objective and specific evaluation methodology. The developmental approach is suitable for evaluating satisfaction with the course. Accordingly, the Dr. LJW Fellowship Program’s training course for health policy administrators adopts Kirkpatrick’s four-level evaluation model, which proposes four levels of program evaluation for an educational module: reaction, learning, behavior, and results to the product phase [13–15]. Participants included 39 public health policy administrators who underwent the Dr. LJW Fellowship training course between 2021 and 2023.

**Figure 2:**
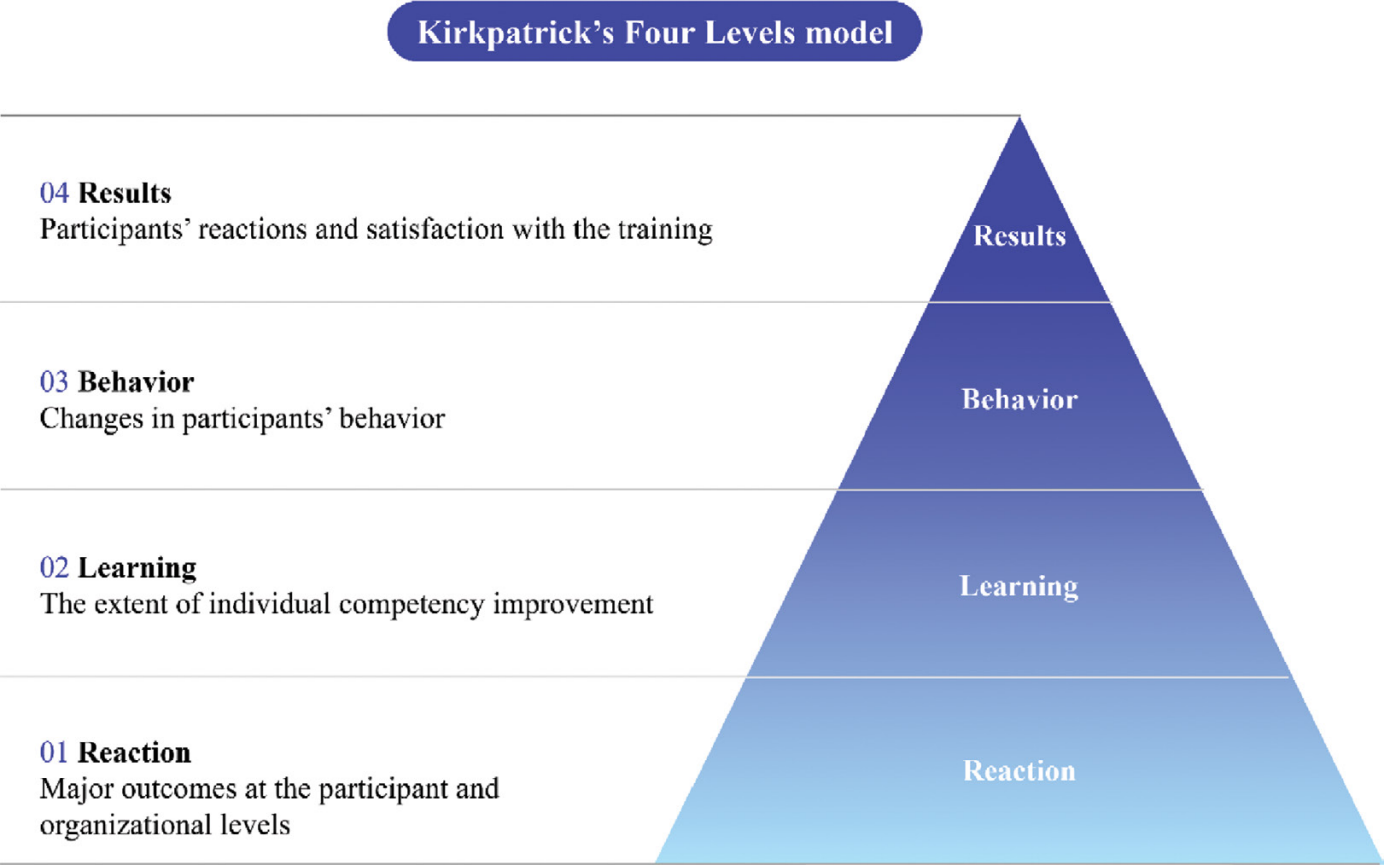
The evaluation model for the Dr. LEE Jong-wook Fellowship Program training course for health policy administrators.

Donald Kirkpatrick developed the Kirkpatrick evaluation model in 1959 to assess training programs and course performance. Kirkpatrick’s model is one of the most widely adopted and frequently referenced frameworks for training evaluation. Known for its simplicity and proven effectiveness over time, it offers a foundational approach to assessing training outcomes. While the model provides limited detail on which specific elements should be evaluated or how to align results with organizational strategy, it serves as a useful guide for the overall evaluation process [15–17]. The model is referred to as “Kirkpatrick’s levels” or the “four levels” because it comprises four evaluation levels (Figure 2).

1. **Evaluation indicators**
  This study highlighted the key indicators as a survey-based evaluation (Supplementary Table 2-5) within Kirkpatrick’s four levels of success that have positive implications for the learning process [18]. The four evaluation levels consist of Level 1 (reaction), Level 2 (learning), Level 3 (behavior), and Level 4 (results) to assess the short-term outcomes of the Dr. LJW Fellowship Program. The final selected indicators comprised six items: training satisfaction, knowledge and competency improvement, competency achievement, competency improvement, work enhancement, and job adoption of learned knowledge. The four levels of the evaluation model and their corresponding indicators are listed in Table 1.
  **Table 1:** The evaluation indicators of this study.

KIRKPATRICK’S FOUR LEVELS	INDICATORS	DESCRIPTION OF INDICATOR	TIME POINT
Reaction	Training satisfaction	**Satisfaction with training content** Evaluates the satisfaction of participants with the goals, content, and environment of the training, as well as their willingness to re-participate, recommends the training, and their awareness of Korea.	At the end of training in Korea
Learning	Knowledge and competency improvement	**Improvement of knowledge and competencies** Evaluates the improvement in participants’ knowledge and competencies through common education, action plan guidance, and Korean language education received during the training in Korea.	Every start/end point of each lecture
	Competence achievement/ improvement	**Achievement of learning objectives** Assesses the achievement of participants in educational and research competencies (knowledge, skills, and attitudes).	2–3 months after the end of training in Korea
Behavior	Work enhancement/job adoption of learned knowledge	**Utilization and transfer of learning outcomes** Assesses, 6 months after the training, whether participants’ job performance has improved due to the training and the extent to which the competencies acquired during the training have been applied in their work.	2–3 months after the end of training in Korea
Results	Self-reported reliability of training and professional enhancement	**Linking training satisfaction to practical application** Self-reported satisfaction to assess the consistency and trustworthiness of course delivery and its relevance to professional roles, alongside the application of acquired knowledge integration.	2–3 months after the end of training in Korea
		The impact of training results in improving the performance of training alumni and/or organizations. Improved performance productivity value-driven practices as part of the learning outcomes.
  The number of items and subcategories is listed in Table 2. The questionnaire demonstrated high reliability across all four indicators. This table presents the internal consistency (Cronbach’s alpha) of the self-administered questionnaire used to assess the four levels of training evaluation indicators: reaction, learning, behavior, and results.
  **Table 2:** Reliability and consistency of the self-administered questionnaire.

INDICATORS	TOOL (LIKERT SCALE)	NO. OF ITEMS (SUBCATEGORIES)	CRONBACH’S ALPHA	RELIABILITY/APPROPRIATENESS
Training satisfaction	5 points	7 (33)	*0.771*	*Highly reliable*
Knowledge and competency improvement	5 points	8 (20)	*0.784*	*Highly reliable*
Competence achievement/ improvement	10 points	2 (3)	*0.857*	*Highly reliable*
Work enhancement/ job adoption of learned knowledge	5 points	2 (10)	*0.805*	*Highly reliable*
2. **Data harmonization and scale standardization of evaluation**
  Evaluation indicators were partially revised each year to reflect the specific professional backgrounds, job positions, and experience levels of the selected fellows. Following the submission of the year-end program report, revisions to both the training curriculum and the evaluation questionnaires continuously refined and disseminated updated evaluation indicators to affiliated training institutions, based on feedback from the external panel committee and KOFIH. Consequently, indices associated with identical evaluation indicators may vary in the structure or scale of their four-level measurements from 2021 to 2023. These Likert-scale-year inconsistencies pose challenges to longitudinal comparisons and program continuity. To address this, the present study standardizes all evaluation indicators by converting them to a 100-point scale. The use of a unified 100-point scale enhances the interpretability of the results and ensures comparability, despite minor changes in the evaluation instruments. This methodological adjustment facilitates a more reliable assessment of the trends in program performance and participant-reported outcomes over time. This approach is consistent with survey research practices in which Likert-type responses are frequently converted to a percentage scale to improve clarity and comparability across instruments and time points [19].
3. **Qualitative evaluation**
  The results component centered on fellows’ self-reported perceptions of training reliability and professional enhancement, gathered through open-ended questionnaires administered online two to three months after program completion (via Zoom meetings and Google surveys). Fellows were invited to articulate how the training influenced their roles and practices in their respective countries and workplaces, adhering to the Consolidated Criteria for Reporting Qualitative Research guidelines. The qualitative design facilitated fellows’ reflections on the impact of the training on their roles and workplaces. The responses were thematically analyzed by two independent researchers utilizing an inductive approach to ensure analytical rigor. These narrative responses provided contextual insights into the real-world impact of the Dr. LJW program and served to complement the quantitative findings.

### Statistical analysis

Descriptive statistics were employed to assess the profiles of participants in the Dr. LJW Fellowship Program training course for health policy administration. Means and standard deviations were calculated for all indicators. Paired t-tests were utilized to compare knowledge and competence before and after the program. The results were derived through a meticulous review of responses collected via Google surveys and participant interviews. Verbalized interview transcripts were coded and analyzed using inductive thematic analysis. Two authors independently coded each transcript to ensure reliability and to capture meaningful user perspectives. The coding framework was refined iteratively through repeated discussions, leading to the development of a set of coherent themes. These final themes were subsequently defined and described, supported by illustrative quotes from participants to provide contextual depth and clarity. Statistical significance was established at *p* < .05 and was deemed significant. Data analysis was conducted using SAS 9.4.

## Results

### Characteristics of participants

Thirty-nine health policymakers participated in the Dr. LJW Fellowship Program training course for health policy administrators from 2021 to 2023; their descriptive statistics are presented in Table 3. Among the participants, 24 (61.5%) were male, and 15 (38.5%) were female. Most participants were in their 30s (59.0%), followed by those in their 40s or older (35.9%), and those in their 20s (5.1%). Their fields of work included chronic disease management (20.5%), personnel management and education (18.0%), maternal and child health (15.4%), health information systems (12.8%), health education (12.8%), disease prevention and health promotion (10.3%), and health management (10.3%). Participants’ work experience was distributed as follows: 6–10 years (38.5%), 11–15 years (28.2%), more than 15 years (20.5%), and 5 years or less (12.8%).

**Table 3:** Characteristics of participants (*n* = 39).

CHARACTERISTICS	*N*, MEAN	%, SD
**Country**		
Ghana	7	17.9
Ethiopia	5	12.8
Uganda	7	17.9
Tanzania	4	10.3
Laos	6	15.4
Cambodia	2	5.1
Vietnam	1	2.6
Indonesia	2	5.1
Uzbekistan	3	7.7
Mongolia	2	5.1
**Age (years, mean)**	**39.1**	**6.2**
**Age group**		
20–29	2	5.1
30–39	23	59.0
Over 40	14	35.9
**Sex**		
Male	24	61.5
Female	15	38.5
**Affiliation**		
Government agency	31	79.5
Public hospital authority and research institute	8	20.5
**Field of work**		
Maternal and child health	6	15.4
Administration of chronic disease	8	20.5
Healthcare information system	5	12.8
Healthcare education	5	12.8
Health prevention and promotion	4	10.3
Healthcare management	4	10.3
Manpower management and education	7	18.0
**Position**		
Top managerial position	5	12.8
Mid administrative position	4	10.3
General administrative position	15	38.5
Professionals	3	7.7
Medical profession	9	23.1
Technical post	3	7.7
**Professional experience (years, mean)**	11.2	5.6
**Professional experience**		
Under 5 years	5	12.8
6–10 years	15	38.5
11–15 years	11	28.2
Over 15 years	8	20.5
**Year**		
2021	12	30.8
2022	13	33.3
2023	14	35.9

*Note*: *N* = number of participants; SD = standard deviation.

### Level 1: Reaction

**Figure 3:**
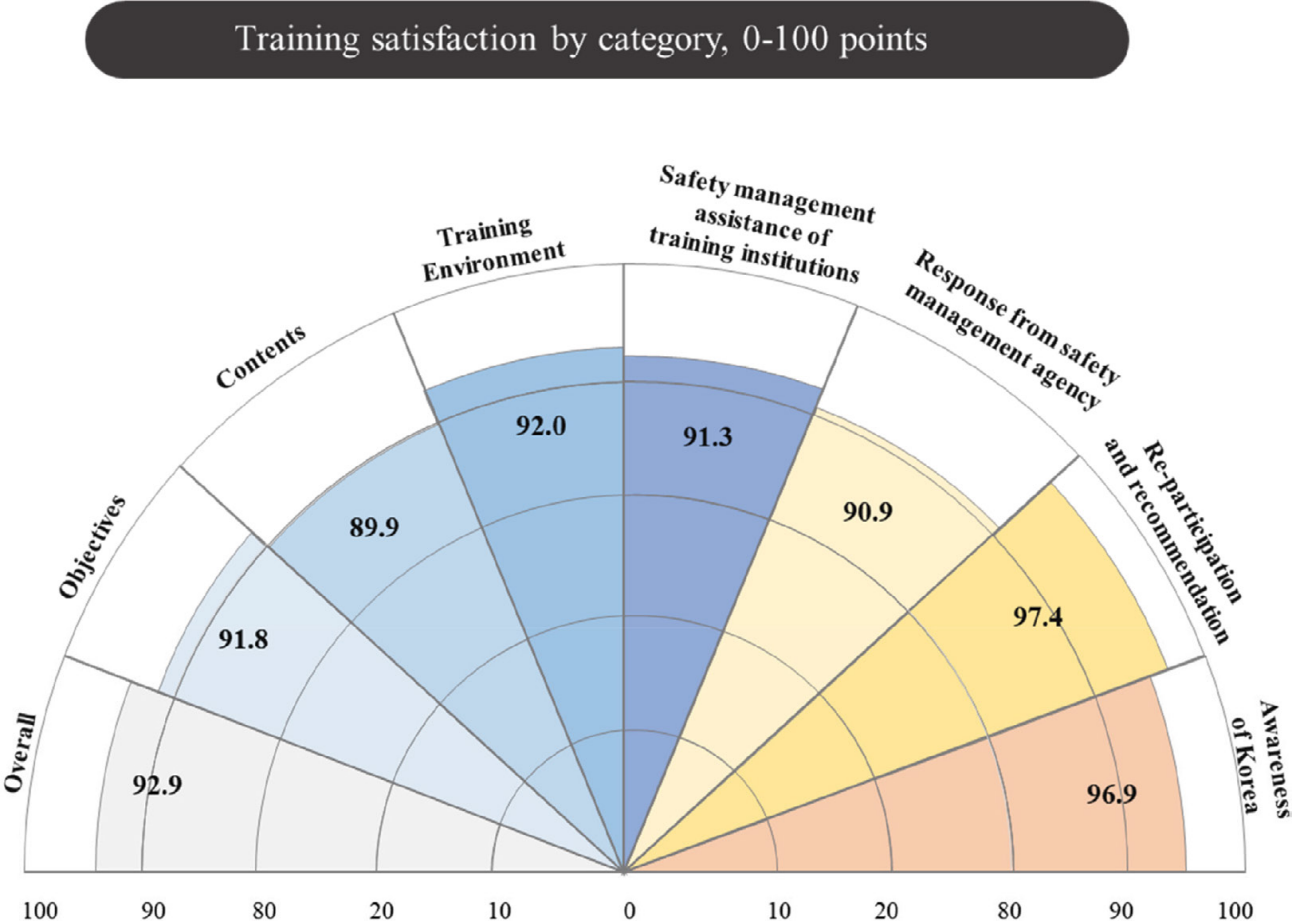
Training satisfaction by category.

1. **Training satisfaction**
  The overall results of training satisfaction are presented in Figure 3. Training satisfaction serves as an indicator that evaluates not only the fellows’ satisfaction with the educational objectives, content, and environment, but also their willingness to participate in the training, recommendations to others, and their awareness of Korea. The mean overall training satisfaction score was 92.9. Satisfaction with the training objectives recorded a mean score of 91.8 (±11.9), while satisfaction with the training curriculum content had a mean score of 89.9 (±8.2). Satisfaction with the training environment was rated at 92 (±7.1). Furthermore, the safety management assistance provided by the training institutions scored 91.3 (±15.1), and the response from the safety management agency was rated at 90.9 (±11.7). The intention to re-participate and recommend the training recorded the highest score at 97.4 (±5.9), while awareness of Korea was rated at 96.9 (±7.3).

### Level 2: Learning

1. **Knowledge and competency improvement**

**Figure 4:**
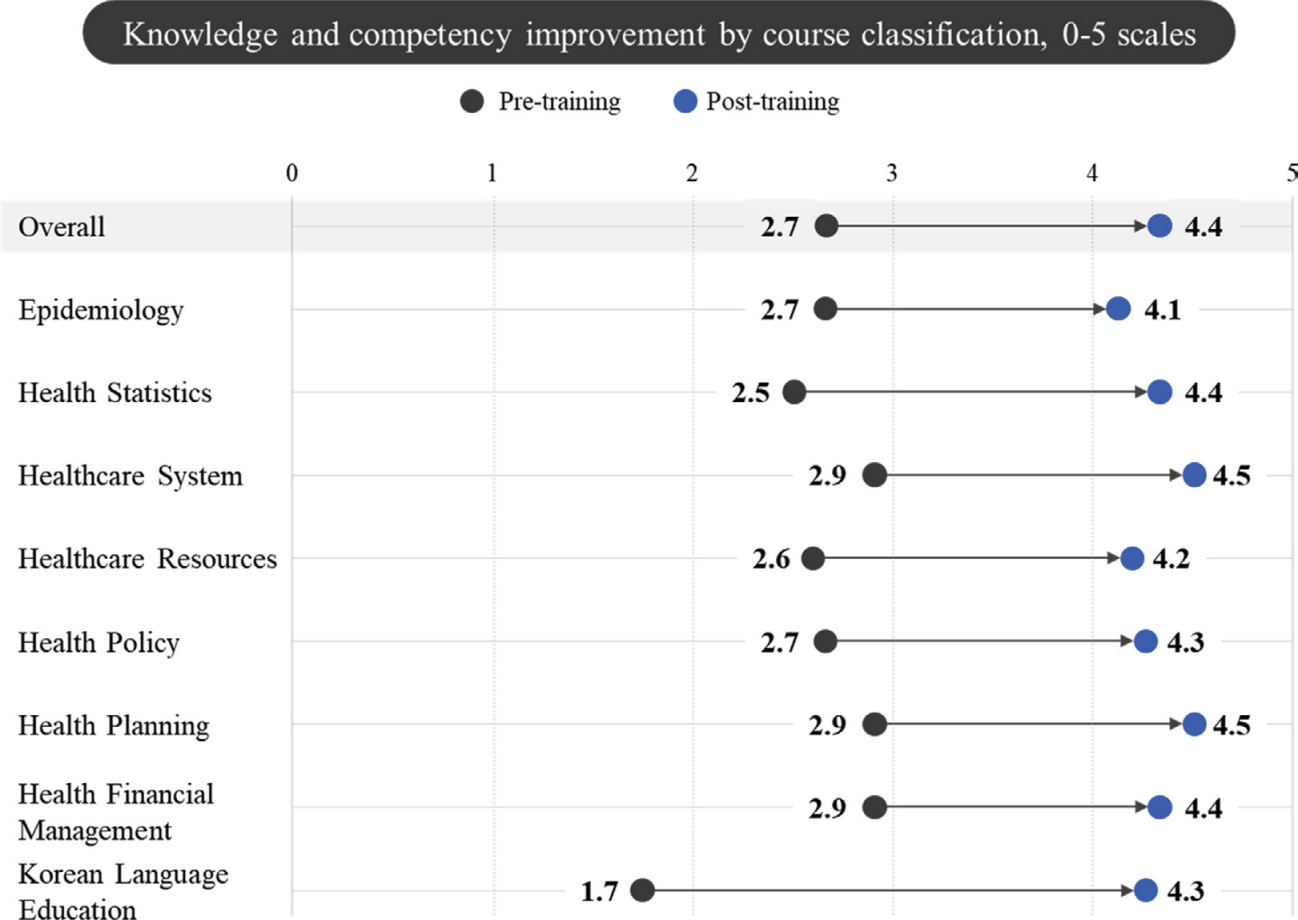
Knowledge and competency improvement by course classification.

The enhancement in knowledge scores across various subjects, evaluated through a comparative analysis of the pre- and post-training scores, is illustrated in Figure 4. Overall, the average score rose from 2.7 prior to the training to 4.4 following the training, indicating a 61.0% improvement. When examining specific subjects, the field of epidemiology demonstrated an increase from 2.7 before the training to 4.1 after the training, resulting in a 48.8% improvement. The health statistics field recorded a noteworthy enhancement, with scores escalating from 2.5 before the training to 4.4 after the training, marking a 77.0% increase. The healthcare systems field exhibited an improvement from 2.9 to 4.5, reflecting a 56.3% increase, while the healthcare resources field showed a 60.8% improvement, with scores rising from 2.6 to 4.2.

In the health policy field, scores increased from 2.7 before the training to 4.3 after the training, resulting in a 60.5% improvement, whereas the health planning field experienced an increase from 2.9 to 4.5, reflecting a 55.2% improvement. The health financial management field also demonstrated progress, with scores ascending from 2.9 before the training to 4.4 after the training, indicating a 51.7% improvement. Finally, the Korean language education field exhibited the highest level of enhancement, with scores rising from 1.7 before the training to 4.3 after the training, resulting in a 150.0% increase.

2. **Competence achievement/improvement**
  Details of the levels of competence achievement during the training period in Korea are presented in Figure 5, demonstrating that the level of competence achievement, as reported by course advisors, had a mean score of 91.0 (±6.2). Conversely, the level of competence achievement, as self-reported by fellows, was slightly higher, with a mean score of 93.9 (±6.7). The average level of competence achievement across these two assessments was 92.5 (±4.4). Furthermore, the overall competence achievement percentage was calculated to be 26.9% (±18.3).
  **Figure 5:**
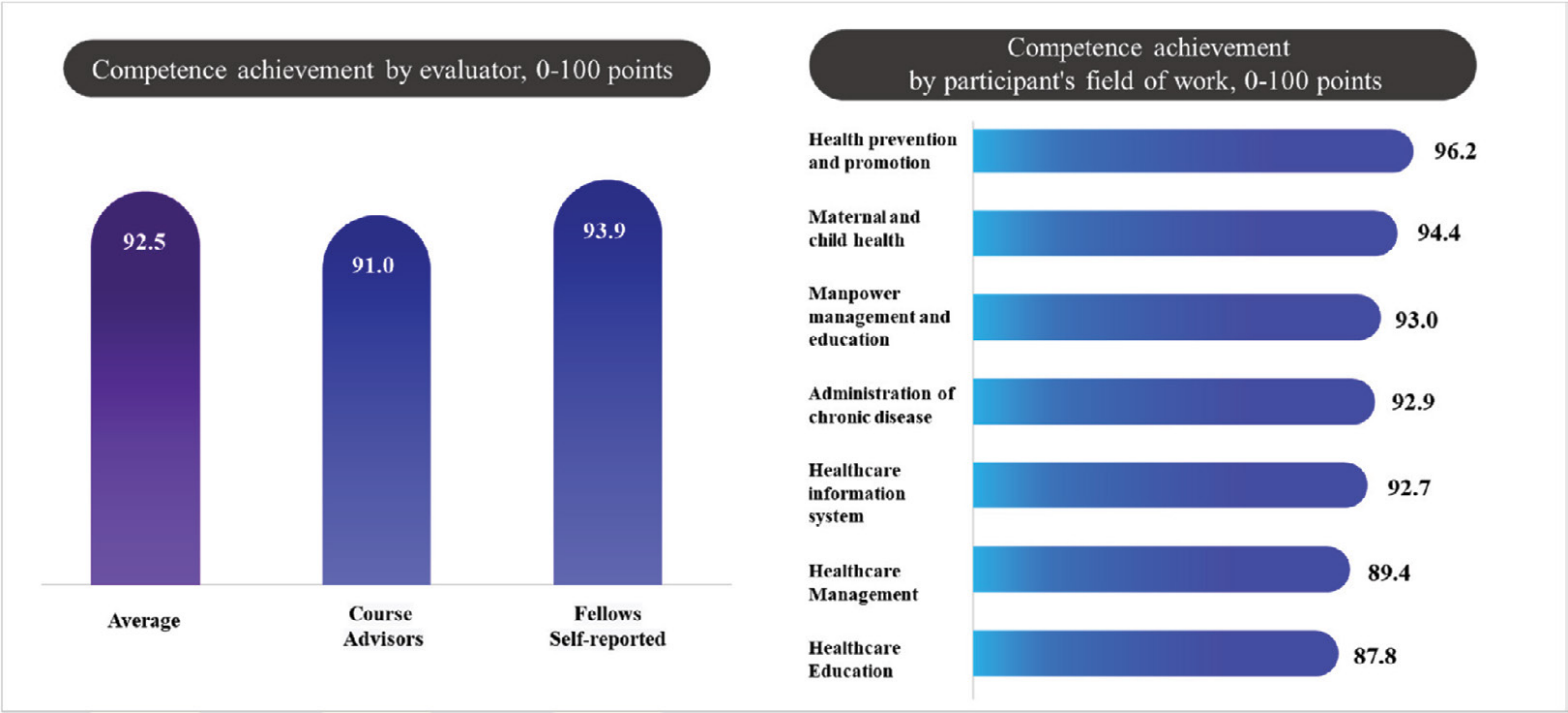
Competence achievement.
  The highest competence achievement was observed in the health prevention and promotion departments, which recorded a score of 96.2. This was followed by the maternal and child health department, which scored 94.4, and the manpower management and education department, which scored 93.0. The administration of chronic disease and healthcare information systems departments demonstrated similar levels of competence achievement, scoring 92.9 and 92.7, respectively. The healthcare management and healthcare education departments exhibited relatively lower competence achievement scores, at 89.4 and 87.8, respectively (Figure 5).

### Level 3: Behavior

1. **Work enhancement**
  The results of work enhancement following the return to work are presented in Figure 6, which displays assessments from the fellows themselves, their supervisors, and coworkers. Specifically, the level of work enhancement, as self-reported by fellows, had a mean score of 90.4 (±9.9). The level of work enhancement, as reported by supervisors, was slightly higher, with a mean score of 92.0 (±9.7). The level of work enhancement assessed by coworkers was 92.2 (±8.8). The average level of work enhancement across all three assessments was calculated to be 91.8 (±7.4).

**Figure 6:**
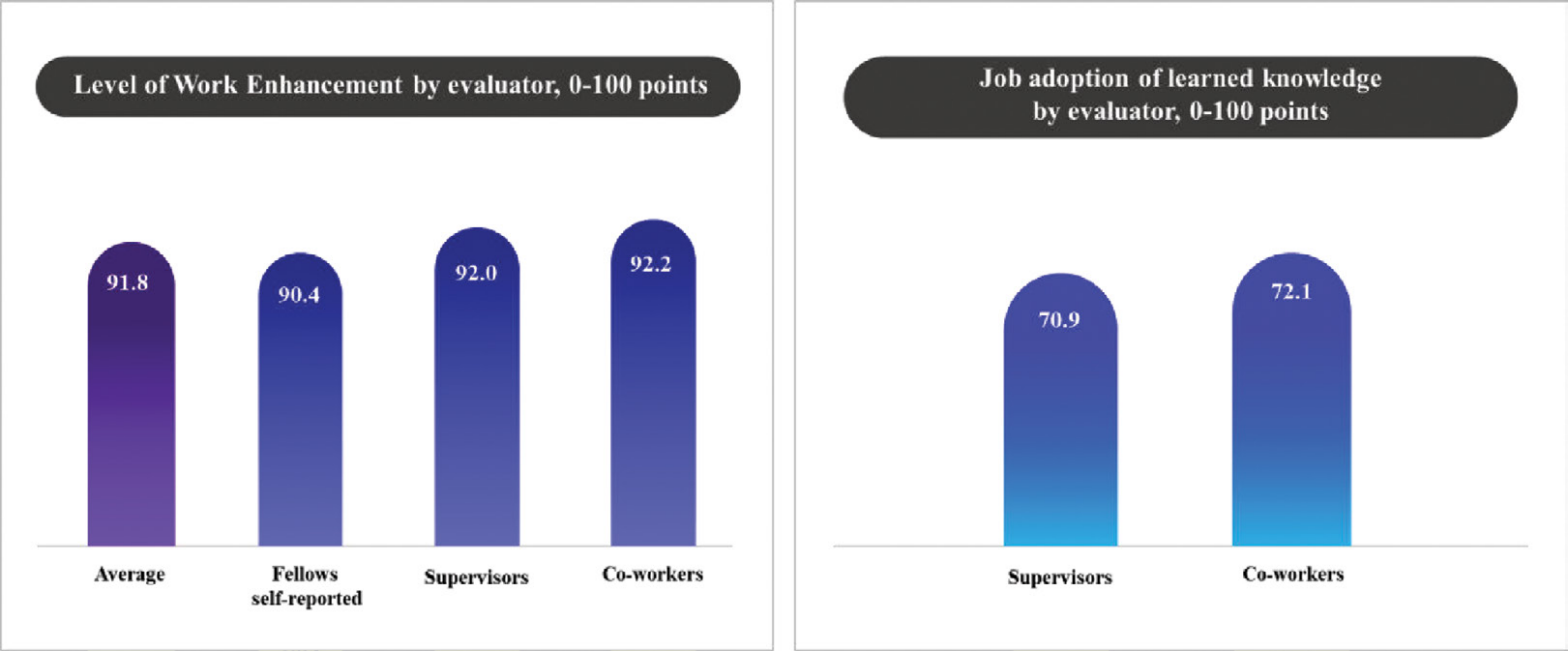
Work enhancement and job adoption.
2. **Job adoption of learned knowledge**
  The results of the evaluation of how well the fellows employed the knowledge gained during the training period in their work, as assessed by their supervisors and coworkers, are shown in Figure 6. Specifically, the level of job adoption of learned knowledge, as assessed by supervisors, had a mean score of 70.9 (±7.0). Meanwhile, the level of job adoption of learned knowledge, as assessed by coworkers, was slightly higher, with a mean score of 72.1 (±6.4).

### Level 4: Results

Level 4 (results) reflects the tangible impact of the training on performance outcomes. Participants reported that the training program significantly enhanced their work productivity and professional competencies. Conversely, fellows were able to deliver outcomes aligned with organizational goals and meet the performance standards and accreditation requirements set by the government. The training contributed to improvements in technical and conceptual skills, effective use of information technology, and adherence to standardized accreditation procedures. The responses of the fellows are transcribed and described in Table 4.

**Table 4:** Self-reported training course satisfaction and professional enhancement of the participants.

INDICATORS	THEME	QUOTE
Reliability of training and professional enhancement	Finance and budgeting	The health financial management and budgeting module provided practical tools that I could immediately integrate into our annual planning cycle. *“Understanding performance-based budgeting and financial forecasting helped me better align resource allocation with our health priorities.”* [#2023]
	Non-communicable diseases (NCDs) policy	The *subject on NCD prevention and health promotion gave me “new strategies for policy formulation at the regional level annual year plan.”* The evidence-based case studies, particularly from other LMICs, were directly applicable to our context. [#2021]
	Hospital/ health technology management	The training and visiting healthcare management and medical technology helped me improve “*how we track and maintain equipment. I was able to propose a digital tracking system for our referral hospital. As a baseline of using laptop for e-health records.”* [#2022]
	Digital health records	The course gave me a foundational understanding of how to design and implement electronic health systems. “*Since returning, I have joined the national task force on digital health and developed electronic medical record system and provided training to the health workers to implement the initial stage of digital system in my health center.”* [#2022]
	NCD awareness and community engagement	The communication tools and behavior change models we learned were exactly what I needed for our diabetes and hypertension awareness campaigns. Most of the community models are incorporated in my project plan. [#2023]
	Emergency and ambulance services	Before the training, I did no t know much about how data linkage or referral systems really worked. But “*the module on the HIRA about national insurance system and how healthcare services are designed and coordinated was very informative. I understood how triage and referral linkages function in practice, and how using data properly can make a huge difference in efficiency that helps to apply in my work.”* [#2023]
	Infectious disease awareness	*“The training deepened my understanding of risk communication and community engagement for infectious disease control, which was not known before training. I have started applying the participatory tools we learned during our outbreak preparedness campaigns.”* [#2021]
	UHC implementation and NHISS planning progress	*“The HIRA training course was highly satisfactory and instrumental in enabling me to independently plan for the implementation of Universal Health Coverage (UHC) and the National Health Insurance Service System (NHISS).”* [#2023] *“The course proved truly valuable for advancing UHC efforts in low- and middle-income countries (LMICs) and helpful to actively participate in Prime minister UHC policy.”* [#2022]
	Enhancement and performance	*“Following the completion of the Fellowship Program, several colleagues have remarked on a noticeable improvement in my work performance, reflecting the program’s positive impact on my professional growth.”* [#2021] *“I believe my professional capacity has improved after completing the training course, I experienced significant professional growth, including improved confidence, enhanced technical skills, and a broader understanding of my field.”* [#2022] *“The course not only deepened my expertise but also motivated me to pursue further innovations and leadership in budgeting and monitoring within my organization.”* [#2023]

*HIRA, Health Insurance Review & Assessment Service; NECA, The National Evidence-based Healthcare Collaborating Agency; KDI, Korea Development Institute; NCC, National Cancer center.*

## Discussion

This project’s overarching goal is to not only strengthen the healthcare delivery system and workforce capacity building of health policy administrators in LMICs but also to improve the health status of vulnerable populations. The Dr. LJW Fellowship Program at KOFIH organized this training course, which will be conducted from 2021 to 2023 at Yonsei University in Seoul. The course curriculum integrates theory, practice, and field trips, while also incorporating visits to Korean cultural sites. Overall, 39 fellows participated in this program, most of them were from Ghana and Uganda, aged 30–40 years, and worked for the Ministry of Health in their home countries. The evaluation of the training course demonstrated excellent levels of satisfaction and acceptability among fellows, including positive feedback from colleagues and superiors, thus indicating the program’s potential for future iterations.

Furthermore, we utilized Kirkpatrick’s year-to model [16, 17, 20, 21] to measure the learner’s obtained knowledge, reactions, and behavior changes [22] in the health information management course [15].

The findings of this study provide valuable insights into the program’s effectiveness, particularly concerning participant satisfaction, knowledge improvement, competence achievement, and the job adoption of learned knowledge. The high levels of satisfaction across various dimensions of the training program, especially regarding reparticipation and recommendations, reflect the program’s strong alignment with the expectations and needs of participants. This alignment is crucial for the program’s sustainability and its potential to attract future cohorts. However, while the satisfaction scores emphasize the program’s effectiveness in providing a conducive learning environment and managing safety, these results should be interpreted with caution, as high satisfaction does not necessarily equate to effective learning or long-term behavioral changes in the workplace, as discussed below.

The learning aspects of the post-training evaluation yielded a higher degree of acquired knowledge and competencies than those observed in the pre-training evaluation. This significant improvement in knowledge scores, particularly in fields such as health statistics and healthcare systems, underscores the program’s success in enhancing participants’ technical expertise. This knowledge gain is not only significant for the individual growth of participants but also for their respective countries, as it may contribute to more informed health policymaking and improved health outcomes at the national level. The high competence achievement scores further support this finding, indicating that participants were able to effectively absorb and apply the training content. Various four-level and training programs have been shown to improve trainees’ knowledge and skills in post-training evaluations in the fields of health policy [23], Internet-based education [24], disease prevention [25], and in-service workforce training [26]. As each country has different goals regarding health issues, advocacy with county health departments and other development partners should prioritize multiple learning methodologies when implementing capacity-building programs.

Additionally, participants’ behavioral changes indicated a greater enhancement of acquired knowledge and skills at work, as evaluated by supervisors and colleagues. Studies have shown that participants report being able to improve their work-related skills and practices through a capacity-building training program, as assessed using an open-ended questionnaire and feedback [27, 28].

However, the relatively lower scores vis-à-vis the job adoption of learned knowledge, as reported by supervisors and coworkers, suggest that while the program is effective in building knowledge and competence, there may have been challenges in translating these gains into practical capacity-building job improvements. This gap between knowledge acquisition and job adoption could be due to various factors, including organizational constraints, cultural differences, and the need for additional support in the home country [26, 27]. These findings indicate that while the program has been successful in many areas, additional interventions may be required to bridge the gap between the knowledge gained during training and its practical application in the workplace.

In addition, the structured qualitative findings highlighted the key areas in which the Dr. LJW Fellowship Program strengthened fellows’ technical and leadership capacities. Participants reported the immediate applicability of training in fields such as budgeting, NCD policy development, digital health records, and emergency service coordination. These findings align with evidence emphasizing the importance of context-specific, practical training for adult learners to drive real-world behavioral change [28, 29].

Beyond individual skill development, fellows have described broader systemic impacts, including enhanced healthcare access strategies, improved infectious disease communication, and revisions to cancer screening programs. These reflections reinforce the role of targeted workforce development programs in supporting on-the-system-strengthening efforts [30, 31]. Additionally, fellows emphasize the credibility and consistency of course delivery, affirming the value of structured mentorship and institutionally anchored training models, as supported by prior studies on global health leadership development [28–32]. The emphasis on structured mentorship and alumni engagement appears critical for fostering sustainable leadership networks in LMIC contexts.

Furthermore, the lower scores regarding the job adoption of learned knowledge, compared to other indicators, highlight the specific difficulties that participants might have faced in integrating their new knowledge into their professional roles. This underscores the need for programs that place greater emphasis on practical applications during the training process, potentially through more hands-on learning opportunities, mentorship, and follow-up support. By addressing these challenges, the program can improve its effectiveness and ensure that participants are better equipped to apply their learning in their respective workplaces.

This study has several limitations. First, although Kirkpatrick’s national-healths were employed, the evaluation may have primarily focused on short-term outcomes, which limits the consideration of long-term behavioral changes and the practical application of skills in the workplace. Moreover, the relatively lower scores for job adoption suggest that the training content may not have been effectively integrated into participants’ professional roles. Given the diverse national contexts of the participants, differences in organizational culture and available resources could have influenced the applicability of the training, potentially limiting the generalizability of the study’s findings. Future studies should consider longer follow-up periods and more culturally sensitive evaluations. Second, the assessments of competence improvement and knowledge application were based on participants’ self-reported responses. Self-reported data may not fully reflect actual improvements in professional competence or the real-world application of skills. Therefore, these findings should be interpreted with caution, and future evaluations should incorporate more objective and validated assessment tools.

## Conclusions

This study demonstrated that the Dr. LJW Fellowship Program training course significantly enhanced participants’ knowledge and competencies. The program effectively assisted health policy administrators in absorbing and applying the training content to their roles. However, the relatively lower scores in the indicate the need for improvement in practical applications. Future programs should strengthen follow-up support to bridge this gap and ensure that participants can effectively integrate what they have learned into their work environments.

## Figures and Tables

**Table:** 

DAC	Development Assistance Committee
Dr. LJW	Dr. LEE Jong-wook
HIRA	Health Insurance Review & Assessment Service
KDI	Korea Development Institute
KOFIH	Korea Foundation for International Healthcare
LMICs	Low- and middle-income countries
NCC	National Cancer Center
NECA	The National Evidence-based Healthcare Collaborating Agency
ODA	Official Development Assistance
OECD	Organization for Economic Cooperation and Development

## Data Availability

The data can be shared upon request after obtaining permission from both the corresponding author and the Korea Foundation for International Healthcare (KOFIH).

## Additional Files

The additional files for this article can be found as follows:

Supplementary File 1. Description of Dr. LEE Jong-wook Fellowship Program.

Supplementary File 2. Survey-Based Evaluation Methodology.
