# Supplementary material for: Enhancing Health Policy Administration in LMICs: Dr. LJW Fellowship Program Insights (2021–2023)
Source: Ann Glob Health. 2025 Jun 18;91(1):34. doi: 10.5334/aogh.4648 (PMC12180440; doi:10.5334/aogh.4648)
Supplement: Supplementary File 1. — Description of Dr. LEE Jong-wook Fellowship Program. [file agh-91-1-4648-s1.pdf]

Supplementary Materials

Supplementary File 1. Description of Dr. LEE Jong-wook Fellowship Program

Supplementary Figure 1. Illustrates the overall program initiation and selection process of host institution. The selected institutions typically administer the same specialized training course for two to three consecutive cycles. This continuity allows for iterative curriculum refinement and programmatic improvement based on accumulated experience. Training institutions fulfill multiple critical functions beyond basic instruction, including candidate assessment through interviews, development of detailed field-specific curricula tailored to participant needs, delivery of comprehensive follow-up support, and collaborative evaluation of training outcomes in partnership with KOFIH.

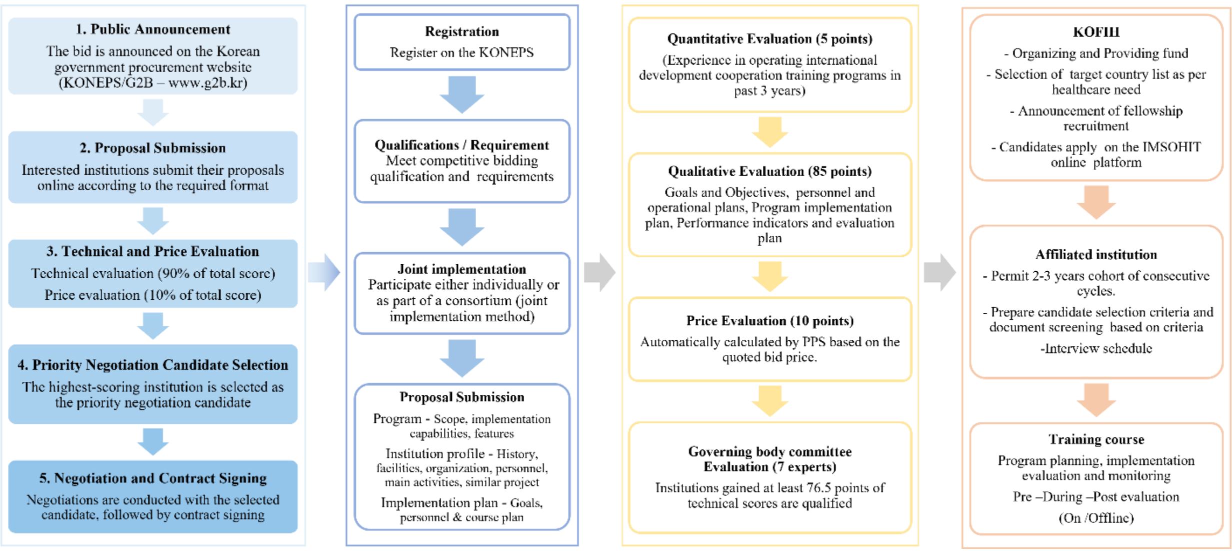

Supplementary Figure 1. Selection and Implementation Process of the Dr. LEE Jong-wook Fellowship program

Program Implementation Stages and Responsibilities

The LEE Jong-wook Fellowship Program follows a structured, three-phase workflow involving multiple stakeholders: KOFIH (Korea Foundation for International Healthcare), designated training institutions, partner countries, and the fellows themselves. Each phase is designed to ensure clarity in responsibilities, alignment with program objectives, and sustainability in post-training impact (see **Supplementary Table 1**).

1. Pre-Training Preparation: KOFIH selects host institutions and coordinates with partner countries, while institutions prepare training materials and partner ministries nominate eligible candidates.
2. Training Implementation: Host institutions deliver academic content and practical modules; fellows participate in structured activities and are supported by academic and mentoring teams. KOFIH ensures training quality and provides diplomatic and technical support.
3. Post-Training Follow-up: Institutions conduct assessments and offer continued academic support. Fellows are encouraged to apply their learning in home country settings, while KOFIH sustains alumni networks and evaluates program impact.

Supplementary Table 1. The detailed structure of the training program

	Module	Category	Main Content
**Module 1**	Pre-training	Orientation	▪ Introduction to the overall curriculum and course syllabus<br>▪ Information on accommodation and daily living
**Module 2**	Training implementation<br>1. Course work	Educational competencies	▪ Courses related to health policy and healthcare management<br>- Epidemiology<br>- Health statistics<br>- Healthcare system<br>- Healthcare resources<br>- Health policy<br>- Health planning<br>- Health financial management<br>- Korean language education
		Participation in international training at the health insurance review and assessment service	▪ Introduction to the main functions and roles of the health insurance Review and assessment service and provision of education for practical application and enhancement of work abilities to contribute to achieving Universal Health Coverage in partner countries
		Conference/academic Meeting participation	▪ Participate in conferences or academic meetings to acquire new knowledge and experience various perspectives through networking with experts
		Korean cultural experience	▪ Traditional tour and cultural experiences to enhance understanding of Korean culture
	2. Individual training	Personalized mentoring	▪ Mentor system between designated instructors and fellows<br>▪ Individual instructors conduct personal interviews with fellows and plan for learning needs based on country-specific health priority strategies and establish customized action plans<br>▪ Develop major learning areas based on fellows' interests
	3. Field visits	Healthcare organization visit	▪ Enhance professional knowledge and skills gained from healthcare facilities and interactions with various human resources.

**Module 3**	Post-training Evaluation and management	▪ Onsite visit<br>▪ Support for On-the-Job Adoption and Consulting<br>▪ Dr. LEE Jong-wook Fellowship health Policy course Exchange meeting	▪ Evaluate workplace knowledge sharing after completing courses at fellows' health facilities, affiliated institutions, and seminars with supervisors and colleagues<br>▪ Monitoring the application of action plans to real-world jobs and addressing challenges<br>- Activities: Conducting action plan presentations and reporting on action plan implementation status, listening to challenges during action plan execution<br>▪ Share cases of on-the-job adoption of returning fellows to enhance networking and performance
