# Supplementary material for: Enhancing Health Policy Administration in LMICs: Dr. LJW Fellowship Program Insights (2021–2023)
Source: Ann Glob Health. 2025 Jun 18;91(1):34. doi: 10.5334/aogh.4648 (PMC12180440; doi:10.5334/aogh.4648)
Supplement: Supplementary File 2. — Survey-Based Evaluation Methodology. [file agh-91-1-4648-s2.pdf]

Supplementary File 2.

Survey-Based Evaluation Methodology

The LEE Jong-wook (LJW) Fellowship Program conducts evaluations throughout the entire program period, from the selection of fellows to the completion of post-training follow-up. These evaluations are based on the *2020 LEE Jong-wook Fellowship Program New Performance Indicator Development Study* and the *Performance Management Guidelines for the LEE Jong-wook Fellowship Program (2022)*. Accordingly, training institutions assess fellows in the areas of reaction, learning, and behavior using predetermined evaluation indicators, tools, and timepoints. Evaluations are conducted at multiple intervals, beginning at the start of the training and extending up to 2 years (typically at around 6 months) including a fellow-up period.

For the Health Policy Administration course, which includes approximately 3 months of training in Korea followed by a post-training management period of 3-4 months, this course evaluated fellows from the start of training until 2-3 months after completion in Korea

Reaction

Training satisfaction

Fellows were assessed in terms of satisfaction with training contexts, the training environment, with the intent of re-participating or recommending the program, and overall awareness of Korea. Specific items included perceptions of training objectives, training content, training environment, training environment, support from the training institution's safety management agency, satisfaction with safety management responses, willingness to re-participate or recommend the program to their supervisors or peers, and awareness of Korea (see **Supplementary Table 2**).

The safety management agency was responsible for matters concerning entry and exit procedures, medical care, and daily life in Korea. However, given the training institution's close interaction with fellows, it played a supplementary role in managing safety-related issues. Scoring for each area was calculated using the following formula:

(Sum of item scores in the post-training satisfaction questionnaire ÷ number of items) ÷ 5 × 100

Supplementary Table 2. The questionaries of training satisfaction

Category	Questionaries
Objectives	Training goals and plans appropriately reflected my initial training needs and goals.
Contents	The training program was helpful in improving my specialty performance.

	The training institution’s training program was useful.
	The contents of the lectures were satisfactory.
	The lecturing methods were satisfactory.
	The difficulty level of training program was appropriate.
	The training period was appropriate.
	Field trips were satisfactory.
	The level of the training program was suitable for my capability.
	Overall, the 2022 HIRA Training course was satisfactory.
Training Environment	Communication with my advisor was smooth.
	My advisor's teaching and advice related to my specialty were useful.
	Communication with Fellow was smooth.
	The educational tools and materials were helpful for training.
	The level of lecture materials was appropriate.
	Training hours per day were appropriate.
	The portion of lectures to the whole program was appropriate.
	Communication in English during the training program was smooth.
	Facilities and environments (classroom, rest area, accommodation, etc.) of training institution were satisfactory.
	Cooking facilities were satisfactory.
	Services provided by Yonsei University (shuttle bus, internet access, etc.) were satisfactory.
	Communication with training institution’s coordinators was smooth.
	Communication in English with the institution's coordinators was smooth.
	The service provided (Lecture notice, Field trip notice, IMSOHIT Assign supports, safety management agency contacts support, etc.) by training institution’s coordinators was helpful.
	The number of Yonsei University' coordinators was sufficient.
	Yonsei University' coordinators were kind.
	The traffic access to the institution was good.
Safety management assistance of training institution	The supporting action in emergency or being sick was efficient.
Response from safety management agency	The supporting action by safety management agency in living was efficient.
	Supports by safety management agency during emergencies or being sick was helpful.
Re-participation and recommendation	I want to recommend the Fellowship to my colleagues in the home country.
	I'm willing to take training in Korea again.
Awareness of Korea	My perception of Korea in the field of my training has improved.

Learning

Knowledge and competency improvement

Knowledge and competency improvement were evaluated through common training modules, Korean language classes, and action plan guidance. Prior to fellows' arrival, training needs assessments were conducted with trainees, their supervisors, and peers. Based on these findings, curricula were developed, and instructors were briefed on the subject's objectives. Evaluation items were designed to measure improvements in knowledge and competency in each subject area.

Fellows completed a pre-evaluation before the first class and a post-evaluation after the final class of each subject. To reduce response bias, fellows who submitted implausible responses (e.g., reporting fluent Korean proficiency despite no prior experience) were asked to revise their answers after clarification. The evaluation items for each subject are provided in **Supplementary Table 3**. Improvement index in each subject area was calculated as follows:

(Average post-evaluation score ÷ average pre-evaluation score) × 100

Supplementary Table 3. The questionaries of knowledge and competency improvement

Category	Subject	Questionaries	Evaluate year
Epidemiology	Seminar in Epidemiology	I understand the national and international health planning perspectives.	2021
		I can explain the characteristics of the Korean communicable disease control system.	2022, 2023
		I can explain the purpose and main steps of epidemiological investigation of communicable diseases.	All
		I can explain the considerations when developing the surveillance & monitoring system.	2021, 2022
		I can explain the characteristics of environmental factors covered by environmental epidemiology.	2022, 2023
		I can explain the exposure assessment method mainly used in environmental epidemiology.	2022
		I understand the interaction between the genetics and environmental factors.	2022, 2023
		I have experience or knowledge about Genetic Epidemiology	2022
		I can explain the purpose of Genetic Epidemiology	2022, 2023
Health Statistics	Biostatistics	I can understand various methods of sampling and describe data with basic statistics in tables and figures	All
		I can understand the concept of statistical inference, point estimation, interval estimation and hypothesis testing.	All
		I can understand R programs and data code.	2022
	Research Methodology	I can understand basic concepts of data analysis and install Jamovi software.	All
		I can identify the form of the given data and create variables as preparations for data analysis.	All
		I can perform statistical analysis directly using exercise data.	All
Healthcare System	Healthcare Delivery System	I can explain the components of the health system.	All
		I can explain the lifelong health care system for each life cycle in Korea.	All
		I can explain the examples of health care systems in underdeveloped countries.	All
Healthcare Resources	Strategic Medical Equipment Management	I understand WHO medical device policies and country-specific primary medical device management systems.	2021, 2022
		I understand medical devices for diagnosis and treatment of COVID-19 and infectious diseases.	2021, 2022
		I know how to use digital health in relation to the 4th industrial revolution and strategies for developing the country's medical device industry.	2021, 2022

	Digital Healthcare in Developing Countries	I have knowledge of the digital healthcare field.	2021, 2022
		I have knowledge of the elements of the e-health framework and analyze the status of each country.	2021, 2022
		I can explain how clinical safety and efficacy can be evaluated.	2021
		I know whether the digital healthcare service guarantees economic feasibility.	2021
		I know whether digital healthcare is an opportunity, or a crisis based on each country's case.	2021
	Quality Management in Healthcare	I can explain the basic concepts of healthcare quality.	2022
		I can explain the variation in medical practice and implications for healthcare quality.	2022
		I can describe the foundation, processes, and tools of healthcare quality improvement.	2022
		I gain a thorough grasp of patient safety and foster enhancement potential.	2023
		I understand the principles of the Patient Safety Reporting and Learning system.	2023
		I understand the notion of patient safety culture.	2023
	Human Resource Management	I can explain how to measure suitability of individuals and organizations in healthcare settings.	2023
		I can explain how to improve job attitude represented by Job Satisfaction.	2023
		I can explain effective work design methods	2023
Health Policy	Global Health Policy and Current Issues	I can understand the management of local health information to strengthen the national health system.	2021, 2023
		I can explain strategies for prevention and treatment of hepatitis to reduce the national burden of disease.	2021, 2022
		I can explain three strategies of Strengthening MCH (Maternal and Child Health) program from the example of Senegal.	2023
		I can explain how far the Sustainable Development Goals are achieved, and strategies to achieve the SDG Goals.	All
		I can explain about patient safety and quality of care evaluation.	2021, 2022
	Health Policy and Practice	I can understand the global trend in nursing education	All
		I can understand nursing education and training in South Korea	All
		I can discuss the challenges for nursing education after COVID-19	All
		I can understand philosophy and principles of person-centered care	2023
		I can describe culture-change movement for person c-centered care across the world	2023

		I can discuss challenges and future directions for implementing person-centered care in long-term care settings	2023
		I can understand philosophy and Principles of Quality End of Life care	2023
		I can describe current trend of end-of-life care across the world	2023
		I can discuss role and leadership in end-of-life care for healthcare reform	2023
	Pharmaceutical Policy of Korea	I can explain the National Health Insurance (NHI), Payment System: Fee-For-Service, and the Pharmaceutical Policy in Korea.	2022, 2023
		I can explain the trend in new drugs, Role of Health Technology Assessment, and the Drug Listing System in Korea.	2022, 2023
		I can explain various Cost-Containment Policies in Korea	2022, 2023
	Ethics of Public Health Practice, Policy, and Research	I can explain the contribution of law for the health of the people.	All
		I can explain the power and limits of state for public health.	All
		I can list the ethical principles of public health practice.	All
		I can explain the importance of ethical consideration in designing, implementing and evaluating public health programs.	All
	National Cancer Control Program	I can explain about the global cancer incidence and mortality, and burden of cancer.	All
		I have knowledge of major cancer management policies to reduce cancer incidence and death.	All
Health Planning	Health planning: Achieving the Sustainable Development Goals	I can describe health planning and its necessary in the healthcare sector.	2021, 2022
		I understand the national and international perspectives of health planning strategies.	2021, 2022
		I can explain in more detailed look at the scientific evidence that human activities are influencing the Earth at the planetary level.	All
		I can understand the concept of sustainability in health planning, its roots, and the sustainable development agenda	2022, 2023
		I can describe the health panning logical model and theory.	2021
		I can describe the SDGs Goal (all about 17 Goals), framework outline in detail and discuss what sets them apart from the previous international guidelines.	2022, 2023
		I can describe the challenges in Information and modern technologies also present unique opportunities for a range of health sectors for health planning	2021, 2023
	Management of Community Health Programs	I can discuss and identify the most important issue(s) to the community.	2022, 2023

		I can explain the focuses on reviewing the evidence base for the program.	2022, 2023
		I have the knowledge, skills and methods for conducting community health assessments and surveillance using health informatics of social and behavioral interventions	2022, 2023
		I can provide practical guidance on how to monitor achievement of a program's objectives	2023
	Monitoring and Evaluation of Health Program	I can explain the role of monitoring and evaluation in public health program planning.	All
		I can describe the elements (inputs, processes, outputs, outcomes) that provide the context for monitoring and evaluation activities.	2021, 2022
		I can describe the primary types, data sources and uses of monitoring systems for developing program indicators.	2021, 2022
		I can explain the evaluation design and its relative strengths and weaknesses.	2022, 2023
		I can develop a monitoring and evaluation plan for the health program	2022
		I can formulate a conceptual framework and logical model of how a program or intervention	2023
	Health System Policy and Health Technology Assessment	I can explain about the types and classifications of health technology.	All
		I can explain about the decision-making on the introduction of health and medical technology and the medical technology evaluation that is the basis for this.	All
		I can explain about the necessity of making policy decisions based on evidence in each country, including Korea.	2021
		I can explain about health care technology, health care system, and policies related to the health care industry.	2021
Health Financial Management	Health Economics	I can understand the characteristics of the health service market	All
		I can understand consumer behavior and decision-making in the healthcare market	All
		I have knowledge of the price elasticity, pricing policies of medical services	2021, 2022
		I can discuss the concept of various costs and cost effectiveness in the healthcare market	All
	Healthcare Financing & Accounting	I can explain that the main contents of accounting and financial management are essential for the operation of health care institutions.	2021
		I can identify key methods for financial decision-making of health care institutions.	2021
Korean Language Education	Korean Language Education	I can say my name, nationality, occupation, and phone number in Korea	2021, 2023

	I can purchase the things and pay for that in Korean	All
	I can order in Korean at the restaurant.	2021, 2022
	I can express my feelings such as pain or unwell symptoms in Korean	All
	When I get lost, I can ask someone by passing by for directions and find my destination.	2021, 2022

Competence Achievement / Improvement

Fellows in the Health Policy Administration course developed an action plan for implementation in their respective workplaces upon completion of training in Korea. During the initial training needs assessment, fellows' job roles, areas of interest, and desired action plans were identified. Based on this, a dedicated advisor was assigned to each fellow. Advisors met with fellows at least biweekly to review and guide the development of their action plans, and to assess competencies in health policy knowledge, skills, and attitudes. At the end of the training, both the advisor and the fellow independently assessed the level of competence achieved. Advisors also evaluated competence improvement by comparing the fellow's baseline (initial meeting) and final performance (see **Supplementary Table 4**). The indexes were calculated using the following formulas:

Competency Achievement = (Advisor evaluation total score ÷ number of items ÷ 5 × 100 × 0.5) + (Fellow self-evaluation total score ÷ number of items ÷ 5 × 100 × 0.5)

Competency Improvement = (Final advisor evaluation score − initial score in the fellow's learning plan) ÷ 10 × 100

Supplementary Table 4. The questionaries of competence achievement / improvement

Category	Evaluator	Questionaries
Competence Achievement	Advisor	His/her knowledge in the relevant field has improved.
		His/her assignment presentation performance was good.
		His/her action plan was good.
		The contents of his/her weekly study report were good.
		He/she actively participated in the training and asked questions.
		He/she diligently performed the assigned tasks.
		He/she diligently attended the entire training course.
		He/she maintained good relationships with professors, colleagues, and staffs.
		He/she can explain the health care system of Korea.
		He/she can explain differences between the health care system of Korea and the health care system of my country.
		He/she has successfully made a plan to apply Korean medical education to my country.
		He/she has taken a serious and open attitude towards Korean health care system.
	Fellow	My knowledge in the relevant field has improved.
		My assignment presentation performance was good.
		My action plan was good.
		The contents of my weekly study report were good.
		I actively participated in the training and asked questions.
		I diligently performed the assigned tasks.
		I diligently attended the entire training course.

		I maintained good relationships with professors, colleagues, and staffs.
		I can explain the health care system of Korea.
		I can explain differences between the health care system of Korea and the health care system of my country.
		I have successfully made a plan to apply Korean medical education to my country.
		I have taken a serious and open attitude towards the Korean health care system.
Competence Improvement	Advisor	Please evaluate the fellow's knowledge and skills at the beginning of the training.

Behavior

Work enhancement / Job adoption of learned knowledge

Upon returning to their workplaces, fellows were expected to share and apply the knowledge and skills gained during their training in Korea. To assess this, surveys were conducted 2-3 months after training completion. Respondents included fellows, their supervisors, and peers. The surveys measured the extent to whether training content was shared, applied in the workplace, and led to observable enhancement in job performance (see **Supplementary Table 5**). The index was calculated as follows:

(Score from ① + ② + ③) ÷ 3, where:

① = (Fellow self-evaluation of work improvement and application) ÷ number of items ÷ 5 × 100

② = (Supervisor evaluation of work improvement and application) ÷ number of items ÷ 5 × 100

③ = (Peer evaluation of work improvement and application) ÷ number of items ÷ 5 × 100

Supplementary Table 5. The questionnaire of work enhancement / job adoption of learned knowledge

Evaluator	Questionaries
Fellow	The Fellowship Program has helped me to gain a new perspective and vision on my field of specialty.
	I am putting the knowledge and skills gained from the Fellowship Program into practical use.
	I think there has been a noticeable improvement in my work performance after the completion of the Fellowship program.
	Colleagues have commented that there has been a noticeable improvement in my work performance after the completion of the Fellowship Program.
	Superiors have commented that there has been a noticeable improvement in my work performance after the completion of the Fellowship Program.
	I am applying what I learned from the Fellowship Program to my work.
Supervisor/ Peer	I think my junior/peer show noticeable improvement in his/her work performance after the completion of the Fellowship program.
	My junior/peer's improved performance had made me interested in the Dr LEE Jong-wook Fellowship Program.
	I think my junior/peer is applying what he/she learned from the Fellowship program to our workplace.
	My junior/peer is educating colleagues and juniors what he has learned to make a difference.
